# Cell Surface Glycan Alterations in Epithelial Mesenchymal Transition Process of Huh7 Hepatocellular Carcinoma Cell

**DOI:** 10.1371/journal.pone.0071273

**Published:** 2013-08-20

**Authors:** Shan Li, Cuiju Mo, Qiliu Peng, Xiaonan Kang, Chun Sun, Kai Jiang, Li Huang, Yu Lu, Jingzhe Sui, Xue Qin, Yinkun Liu

**Affiliations:** 1 Department of Clinical Laboratory, First Affiliated Hospital of Guangxi Medical University, Nanning, Guangxi, People's Republic of China; 2 Cancer Research Center, Institute of Biomedical Science, Fudan University, Shanghai, People's Republic of China; 3 Liver Cancer Institute, Zhongshan Hospital, Fudan University, Shanghai, People's Republic of China; The University of Hong Kong, China

## Abstract

**Background and Objective:**

Due to recurrence and metastasis, the mortality of Hepatocellular carcinoma (HCC) is high. It is well known that the epithelial mesenchymal transition (EMT) and glycan of cell surface glycoproteins play pivotal roles in tumor metastasis. The goal of this study was to identify HCC metastasis related differential glycan pattern and their enzymatic basis using a HGF induced EMT model.

**Methodology:**

HGF was used to induce HCC EMT model. Lectin microarray was used to detect the expression of cell surface glycan and the difference was validated by lectin blot and fluorescence cell lectin-immunochemistry. The mRNA expression levels of glycotransferases were determined by qRT-PCR.

**Results:**

After HGF treatment, the Huh7 cell lost epithelial characteristics and obtained mesenchymal markers. These changes demonstrated that HGF could induce a typical cell model of EMT. Lectin microarray analysis identified a decreased affinity in seven lectins ACL, BPL, JAC, MPL, PHA-E, SNA, and SBA to the glycan of cell surface glycoproteins. This implied that glycan containing T/Tn-antigen, NA2 and bisecting GlcNAc, Siaα2-6Gal/GalNAc, terminal α or βGalNAc structures were reduced. The binding ability of thirteen lectins, AAL, LCA, LTL, ConA, NML, NPL, DBA, HAL, PTL II, WFL, ECL, GSL II and PHA-L to glycan were elevated, and a definite indication that glycan containing terminal αFuc and ± Sia-Le, core fucose, α-man, gal-β(α) GalNAc, β1,6 GlcNAc branching and tetraantennary complex oligosaccharides structures were increased. These results were further validated by lectin blot and fluorescence cell lectin-immunochemistry. Furthermore, the mRNA expression level of *Mgat3* decreased while that of *Mgat5*, *FucT8* and *β3GalT5* increased. Therefore, cell surface glycan alterations in the EMT process may coincide with the expression of glycosyltransferase.

**Conclusions:**

The findings of this study systematically clarify the alterations of cell surface glycan in cancer EMT, and may provide novel insight for HCC metastasis.

## Introduction

Hepatocellular carcinoma (HCC) is the fifth most common cancer widespread and ranks third cancer mortality globally [1]. The high mortality is caused by tumor recurrence, metastasis and lack of effective therapeutic response. Metastasis is the major obstacle of HCC treatment efficacy, so it is urgent to discover better biomarkers that predict HCC metastasis. Recent studies demonstrated that epithelial-to-mesenchymal transition (EMT) is a critical step of tumor metastasis [2]. EMT is a developmental process during which cells switch from an epithelial phenotype to a mesenchymal phenotype and gain the ability to migrate. This process accompanied by the down-regulation or loss of the epithelial cell markers, such as E-cadherin, cytokeratin, mucin; and up-regulation of mesenchymal markers, such as N-cadherin, vimentin, fibronectin, transcription factors snail and slug, etc [3]. EMT phenomenon was discovered in the cells, animal models and clinical studies of HCC; the occurrence of EMT was related to the metastasis and chemotherapy drug resistance of HCC [4]. In human HCC, EMT is highly correlated with invasive tumors, intrahepatic metastasis, and poor survival [5]. As well known a wide variety of growth factors including transforming growth factor β(TGF-β), hepatocyte growth factor (HGF), epidermal growth factor (EGF), fibroblast growth factor (FGF) could up-regulate the expression of EMT-regulating transcription factors [6], ultimately promoting the occurrence of EMT. In this study, we used HGF induced EMT of human Huh7 HCC cells.

Glycosylation is the most common post-translational modification. The carbohydrate structures of cell surface glycoconjugates play an important role in many physiological and pathological events, including cell growth, differentiation, and transformation [7]. Many studies have reported that protein glycosylation was related to tumor development, invasion and metastasis, and found that characteristic glycoprotein biomarker showed an increasingly important role in diagnosis. Some glycoproteins with aberrant glycosylation have been used as biomarkers for HCC diagnosis, such as AFP-L3 [8], aleuria aurantia lectin (AAL)-reactive α1-antitrypsin [9], α-1,3 fucosylated multiantennary glycan on hemopexin [10]. But there were poor glycan of glycoprotein as biomarkers for HCC metastasis. Therefore, studies focusing on dynamic glycoproteomic alteration during cancer metastasis can help to clarify the mechanism of HCC metastasis and may provide potential markers for HCC metastasis.

In this study, we used HGF-induced HCC EMT model in vitro, and then analyzed the profile of cell surface protein glycosylation by lectin microarray. Alterations of cell surface glycoconjugates on benefit for tumor cells to escape from immune surveillance as well as metastasis [11]. The goal of this study was to profile HCC metastasis-specific, lectin reactive glycan of glycoproteins with comparisons to that of its primary cancer. It may provide the critical insight for the effective control of HCC metastases and improve patient survival rate.

## Materials and Methods

### Cell culture

The human HCC Huh7 cells (obtained from Cell Bank, Chinese Academy of Sciences, Shanghai, China) were maintained in Dulbecco's modified Eagle's (DMEM) medium (Sigma, USA) supplemented with 10% fetal bovine serum (FBS, Gibco, USA), 100 U/mL streptomycin, and 100 U/mL penicillin in a humidified atmosphere of 5% CO2 at 37°C. A total of 10^5^ cells were seeded in 6-well tissue culture dishes. After 24 h of incubation, the cells were treated with or without HGF (R&D Systems, USA) in serum-free DMEM with daily replacement of the culture medium. HGF was stored in PBS containing 0.1% BSA and added into the serum-free DMEM to a final concentration. Cell morphology was observed by Inverted phase contrast microscope.

### Quantitative real-time reverse transcription-polymerase chain reaction (qRT-PCR)

Total RNA was isolated using Trizol reagent (Invitrogen) according to the Manufacturer's instructions. Two micrograms of RNA were reverse-transcribed using a reverse transcriptase reaction kit (Ferments). Taq DNA polymerase was purchased from TAKARA. PCR was performed using SYBR Green PCR Master Mix and reactions were carried out on IQ5 Multicolor Real-time PCR Detection System (Bio-Rad) with the amplified conditions: 95°C for 5 min, 40 cycles of 95°C for 15 s, 60°C for 15 s and 72°C for 20 s. The relative expression value was calculated by 2^−ΔΔT^ method. The primer sequences were listed in Table 1.

**Table 1:** List of primers used for qRT-PCR.

Gene	Primer sequences
*E-cadherin*	Forward: 5′-GAATGACAACAAGCCCGAAT-3′Reverse: 5′-GACCTCCATCACAGAGGTTCC-3′
*N-cadherin*	Forward: 5′-GGTGGAGGAGAAGAAGACCAG-3′Reverse: 5′-GCATCAGGCTCCACAGT-3′
*Slug*	Forward: 5′-TGGTTGCTTCAAGGACACAT-3′Reverse: 5′-GTTGCAGTGAGGGCAAGAA-3′
*Snail*	Forward: 5′-GCTGCAGGACTCAATCCAGA-3′Reverse: 5′-ATCTCCGGAGGTGGGATG-3′
*Twist*	Forward: 5′-CCCAACTCCCAGACACCTC-3′Reverse: 5′-CAAAAAGAAAGCGCCCAAC-3′
*Mgat3*	Forward: 5′-GCCTCACCTTGGGAGTTATC-3′Reverse: 5′-GCATCATTGGGTAGCGTCTG-3′
*Mgat5*	Forward: 5′- GCTGCCCAACTGTAGGAGAC-3′Reverse: 5′- GAATCAAGGACTCGGAGCAT-3′
*β3GalT5*	Forward: 5′- CAGATAACCCGTGGGGATAG-3′Reverse: 5′- GCACCAAGTGGGAACTAATC-3′
*FUT8*	Forward: 5′- AGCGAACACTCATCTTGGAA-3′Reverse: 5′- TTGACAAACTGAGACACCCA-3′
*β-actin*	Forward: 5′- CATGTACGTTGCTATCCAGGC -3′Reverse: 5′- CTCCTTAATGTCACGCACGAT-3′

### Western blot analysis

Cell lysis buffer was purchased from Beyotime (P0013) and stored at −20°C until assay. The protein concentrations were determined with the bicinchonininc acid (BCA) method. Equivalent aliquots of 20 μg proteins were separated by 10% SDS-PAGE and transferred onto polyvinylidene fluoride membrane (PVDF,Millipore, Billerica, USA) using a Bio-Rad SemiDry instrument. After blocking for nonspecific binding with 5% non-fat dry milk, the membrane was incubated with antibodies against E-cadherin (BD Bioscience, 1∶1000 dilution), N-cadherin (BD Bioscience, 1∶2000 dilution), vimentin (Abcam,1∶25 dilution), α-SMA (Abcam,1∶400 dilution), Snail (Abcam,1∶1000 dilution), and GAPDH (KangChen Bio-tech, 1∶10000 dilution) respectively at 4°C overnight. The membrane was washed three times with TBST (50 mmol/L Tris-HCl, 150 mmol/L NaCl, 0.1% v/v Tween-20, pH 7.4) and incubated with HRP-conjugated secondary antibodies (1∶10000 dilution) for 1 h at room temperature. After washing three times with TBST, the protein bands were visible and semi-quantitative analysis using enhanced chemiluminescence detection (ECL; GE, Healthcare, Piscataway, NJ).

### Cell migration assays

Cell migration was performed using transwell chamber with 8 μm pores (Corning Costar, Cambridge, MA). Huh7 cells treated with 10 ng/ml HGF for 72 h were trypsinized and resuspended in DMEM containing 3% FBS. Cells (5×10^4^) were plated in the upper chamber, and the upper chambers were inserted in a well of a 24-well plate containing 600 μl DMEM containing 10% FBS. After incubation for 36 h, the non-migrating cells in the upper chamber were removed using a cotton swab and the cells that had migrated to the underside of the membrane were fixed with 4% paraformaldehyde for 30 min, and then stained with 10% Giemsa for 30 min. The cells in the underside of the membrane were counted under light microscopy. Huh7 cells were cultured in serum-free DMEM without HGF stimulation for 72 h as control.

### Cell adhesion assay

Matrigel was formulated as a 10 μg/250 μl concentration with serum-free DMEM medium, then the 96-well plates were put in the clean bench for seasoning overnight after adding 2 μg/50 μl matrigel per well. The plates were washed with PBS to remove undesired matrigel. HGF-induced and control cells were collected and resuspended in serum-free DMEM medium. A total of 4×10^3^ cells were seeded in each well with three replications hole, and plates were incubated for 20 min, 40 min and 60 min at 37°C in 5% CO_2_. Unattached cells were washed away by PBS for three times. The attached cells were fixed with methanol for 15 min, and then stained with Giemsa for 15 min. The number of adherent cells was counted under a microscope after washing with water.

### Cell lectin microarray

Cells were washed in PBS and collected after trypsin treatment by centrifugation at 125 g for 5 min. The cell pellets were immobilized by 3% glutaraldehyde for 15 min and labeled by Cy5 Mono-Reactive Dye Pack (GE, USA) in 0.1 M sodium bicarbonate buffer (pH 9.3) for 1 h at room temperature in dark condition. In this process, it is necessary to stir the cells every 10–20 min to prevent them from clumping together. After washing in PBS twice, cells were resuspended at 2×10^6^ cells/ml in 0.1 M sodium bicarbonate buffer (pH 9.3) for backup.

The lectin microarray was prepared as previous publication [12]. Lectins were purchased from Vector. Total 50 kinds of tumor-associated lectins were dissolved in 2% BSA–TBS solution (pH 7.8) at a concentration of 1 mg/ml and spotted with six repeats on hydrogel slides (CapitalBio, Beijing, China) using Smart Arrayer-8 (CapitalBio, Beijing, China). There were 400 mm between spots, and the diameter of each spot was 150 mm. After spotting, for the purpose of immobilizing the lectins, the hydrogel slides were incubated in a vacuum chamber at 25°C overnight. The lectins and their specific binding carbohydrates were shown in Table 2. For blocking the non-specific binding sites, the lectin microarray was treated with 2% BSA–TBS at room temperature for 1 h. After washing for three times in 0.1% PBS-Tween20, the labeled cells were added to each well (100 μl/well) and incubated at room temperature with gentle shaking for 3 h. The lectin microarray was washed in 0.1% PBS-Tween20 for 5 min three times to separate the unbound cells. The lectin microarray was detected and the data was extracted by a LuxScan 10 K/A scanner system (CapitalBio). The fluorescence intensities of each spot were calculated by subtracting background from signal intensity. The Grubbs' outlier method was used to statistically test repeat data, and the data of different microarrays were normalized by median. T-test was used to statistically analyze the fluorescence intensity of each kind of lectin spots binding different samples. The difference was statistically significant for P<0.05.

**Table 2:** Lectins used in the array and their specific binding carbohydrates.

Number	Lectins	specific binding carbohydrates
**1**	Aleuria aurantia lectinn (AAL)	Terminal αFuc and ± Sia-Le
**2**	Agaricus bisporus lectin (ABL)	Galβ1-3GalNAcα1-Ser/Thr (T-Antigen),Siaα2-3 (6) Galβ-1-3GalNAcα1-Ser/Thr
**3**	Amaranthus caudatus lectin (ACL)	galactosyl (b-1,3) N-acetylgalactosamine structure
**4**	Bauhinia purpurea lectin (BPL)	galactosyl (b-1,3) N-acetylgalactosamine structures
**5**	Bandeiraea simplicifolia lectin (BSL)	α-D-galactosyl residues, N-acetyl-α-D-galactosaminyl residues
**6**	Caragana arborescens lectin (CAL)	ForssmanpentasaccharidE
**7**	Codium fragile lectin (CFL)	N-acetyl-D-galatosamine
**8**	Concanavalin A (Con A)	α-man (inhibited by presence of bisecting GlcNAc) biantennary complex-type oligosaccharides
**9**	Cytisus scoparius Lectin (CSL)	D-galactose and by N-acetyl-D-galactosamine
**10**	Dolichos biflorus agglutinin (DBA)	a-linked N-acetylgalactosamine
**11**	Datura stramonium agglutinin (DSA)	>Biantennary,(G1cNAc)n,polyLacNAc and LacNAc (1NA3,NA4)
**12**	Erythrina cristagalli lectin (ECL)	galactosyl (b -1,4) N-acetylglucosamine
**13**	Euonymus europaeus lectin (EEL)	galactosyl (a-1,3) galactose
**14**	Galanthus nivalis lectin (GNL)	(a-1,3) mannose residues
**15**	Griffonia simplicifolia lectin I (GSL I)	a-N-acetylgalactosamine residues, a-galactose residues
**16**	GSL I – isolectin B4 (GSL1b4)	a-N-acetylgalactosamine residues, a-galactose
**17**	Griffonia simplicifolia lectin II (GSL II)	alpha- or beta-linked N-acetylglucosamine residues
**18**	Helix aspersa lectin (HAL)	terminal N-acetyl-α-D-galactosaminyl residues
**19**	Hippeastrum hybrid lectin (HHL)	(a –1,3) and (a–1,6) linked mannose structures
**20**	Helix pomatia lectin (HPL)	α-N-acetyl-D-galactosamine
**21**	Jacalin (JAC)	galactosyl (b–1,3) N-acetylgalactosamine
**22**	Lens culinaris agglutinin (LCA)	Fucα1–6GlcNAc and α-Man,α-Glc,GlcNAc–Asp of the trimannosyl core
**23**	Lycopersicon esculentum lectin (LEL)	N-acetylglucosamine oligomers
**24**	Limulus polyphemus lectin (LPL)	N-acetylated D-hexosamines
**25**	Lotus tetragonolobus lectin (LTL)	alpha-linked L-fucose
**26**	Maackia amurensis lectin I (MAL I)	Siaα2-3Gal
**27**	Maackia amurensis lectin II (MAL II)	Siaα2-3Gal
**28**	Maclura pomifera lectin (MPL)	Galβ1–3GalNAcα-Ser (T) and GalNAcα–Thr/Ser (Tn)
**29**	Naja mossambica lectin (NML)	like ConA, exopolysaccharide
**30**	Narcissus pseudonarcissus lectin (NPL)	polymannose structures containing (a-1,6) linkages.
**31**	Peanut agglutinin (PA)	galactosyl (b-1,3) N-acetylgalactosamine
**32**	Phytolacca americana lectin (PAL)	β-GlcNac
**33**	Pseudomonas aeruginosa lectin (PAL)	D-galactose
**34**	Phaseolus coccineus lectin (PCL)	sialic acid
**35**	Phaseolus vulgaris Erythroagglutinin (PHA-E)	NA2 and bisecting GIcNAc
**36**	Phaseolus vulgaris Leucoagglutinin (PHA-L)	Tetraantennary complex oligosaccharides
**37**	Pisum sativum agglutinin (PSA)	a-linked mannose-containing oligosaccharides
**38**	Psophocarpus tetragonolobus lectin I (PTL I)	alpha linked galactosamine
**39**	Psophocarpus tetragonolobus lectin II (PTL II)	beta anomeric configuration
**40**	Ricinus communis agglutinin I (RCA I)	Lac/LacNAc,Terminal Galβ 1-4 GlcNAcβl
**41**	Ricin B Chain (RIC)	Galactose/N-acetylgalactosamine
**42**	Soybean agglutinin (SBA)L	terminal a- or b-linked N-acetylgalactosamine
**43**	Sophora japonica agglutinin (SJA)	N-acetylgalactosamine and galactose residues
**44**	Sambucus nigra lectin (SNA)	Siaα2–6Gal/GalNAc
**45**	Solanum tuberosum lectin (STL)	oligomers of N-acetylglucosamine
**46**	Ulex europaeus agglutinin (UEA)	a -linked fucose residues
**47**	Viscum album lectin (VAL)	β-D-galactosyl residues
**48**	Vicia villosa lectin (VVL)	alpha- or beta-linked terminal N-acetylgalactosamine
**49**	Wisteria floribunda lectin (WFL)	N-acetylgalactosamine linked alpha or beta to the 3 or 6 position of galactose
**50**	Wheat germ agglutinin (WGA)	(GlcNAc)n,multivalent Sia and GalNAc

### Lectin blot

To identify and validate the results of the lectin microarray, we carried out the lectin blot experiment. Membrane proteins were extracted by ProteoExtractTM Native Membrane Extraction Kit (Merk, Germany) according to the manufacturer's instruction. Firstly, cells were washed with ice cold wash buffer. Secondly, the protease inhibitor cocktail and extraction buffer I were added to the wall of the cell container, the cells were incubated for 10 min at 4°C under gentle agitation. The supernatant was discarded. Last, the protease inhibitor cocktail and extraction buffer II were added to the wall of the cell container, the cells were incubated for 30 min at 4°C under gentle agitation. The supernatant with enriched membrane proteins was transferred into a sample tube for follow-up experiments.

Membrane proteins were further separated by 10% SDS-PAGE and then transferred onto PVDF membrane. The membrane was stained in accordance with the MemcodeReversible Protein Stain Kit (Pierce, USA) to contrast the same injection volume of protein. And it was used as the internal control. The PVDF membrane was blocked with 2% BSA-TBST for 1 h and then incubated with biotinylated lectins at room temperature for 30 min. After washing with TBST four times, the PVDF membrane was then incubated with avidin D-HRP at room temperature for 30 min, and washed with TBST four times. The bands on the membrane were detected using enhanced chemiluminescence detection.

### Fluorescence cell lectin-immunochemistry

After 72 h incubation, the HGF-treated Huh7 cells and control cells were seeded into a 24-well plate. After washing with PBS, cells were fixed with methanol for 15 min. Then biotinylated lectins were added to each well and incubated at room temperature for 30 min under gentle agitation. Then added Streptavidin-Alexa fluor 488 (Molecular Probes) incubated for 30 min at room temperature after washing for 3 times. Nuclei were stained by DAPI (Sigma) at room temperature for 5 min. After the final washing, the 24-well plate was observed and photographed by fluorescence microscope.

### Statistical analysis

The statistical analyses were performed using a commercially available statistical software package (SPSS for Windows, 16.0). Quantitative variables were analyzed by Student's t-test. P<0.05 was considered statistically significant.

## Results

### Characteristics of EMT in HGF induced Huh7 cells

**Figure 1:**
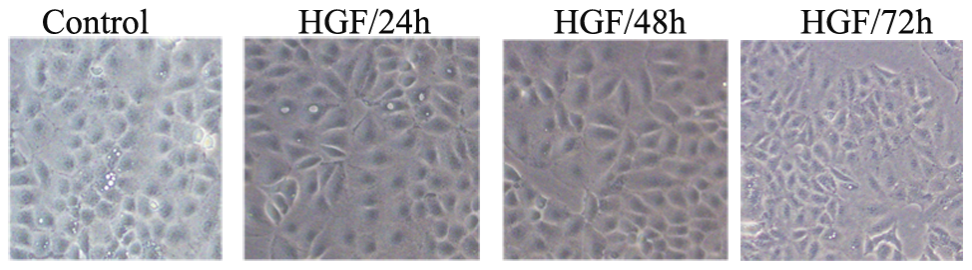
Morphological changes after HGF treatment in Huh7 cells (200×).

After treatment with HGF, Huh7 cellular morphology was converted to a diffused fibroblast-like morphology, presented as the characteristic of EMT, as compared with untreated cells (Figure 1). A few of cells shape elongated with HGF treated 24 h, after 48 h or 72 h, cellular morphology changed obviously.

**Figure 2:**
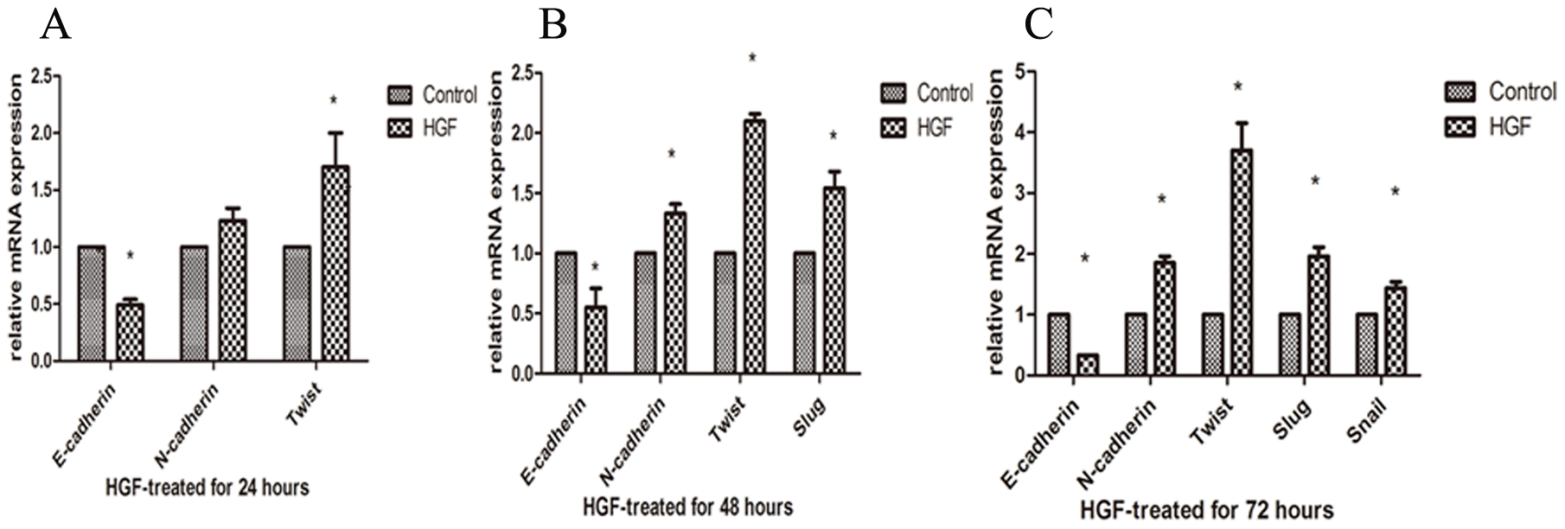
Changes in the mRNA expressions of the EMT-related genes induction by 10 ng/ml HGF (24 h, 48 h, 72 h) analysis by qRT-PCR. All of the relative expression levels of EMT-related genes normalized to β-actin. *p<0.05.

**Figure 3:**
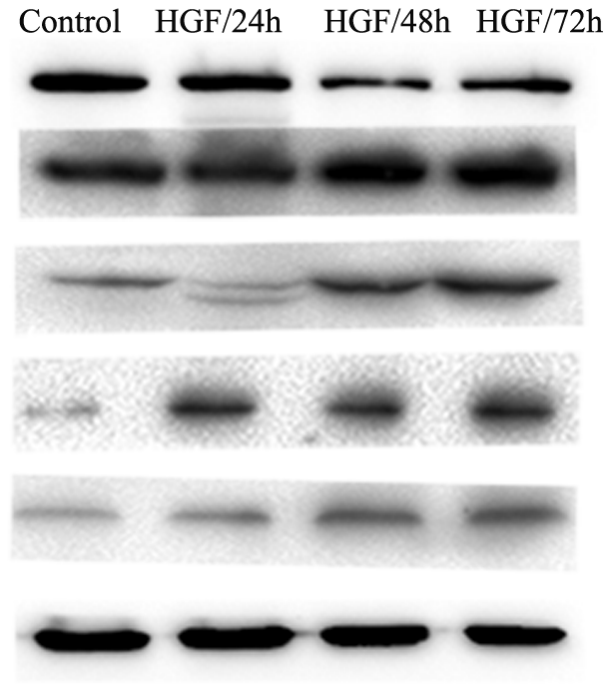
Expression of EMT-associated proteins treated with 10 ng/ml for the indicated time course (0, 24, 48, or 72 h) was determined using a western blot analysis.

In order to further investigate HGF can induce EMT in Huh7 cells; here we examine the more common markers of EMT, using qRT-PCR and western blot. All of the data results were from at least three independent experiments. E-cadherin was the prototypical epithelial cell marker; the cadherin switch from E-cadherin to N-cadherin have a profound effect on the progress of EMT during cancer progression [13]. Our data showed down-regulation of E-cadherin and up-regulation of N-cadherin both in gene and protein by HGF stimulation (Figure 2 and Figure 3). Transcription factors, Slug, Snail, Twist, play an important role in the process of EMT through the cadherin switching to promote tumor cells invasion and metastasis. As the data shows, Slug, Snail, and Twist are also up-regulated in mRNA or protein expression level after HGF stimulation (Figure 2 and Figure 3). Vimentin existed in mesenchymal cells and was a marker commonly used to identify cancer cells in the EMT process. α-SMA expressed in myofibroblasts, can be used as EMT indicators. They also increased in levels of protein as mesenchymal markers (Figure 3). To sum up, HGF-induced HCC EMT model was successfully established in vitro.

### Enhanced invasive ability and weakened adhesion of cells treated with HGF

**Figure 4:**
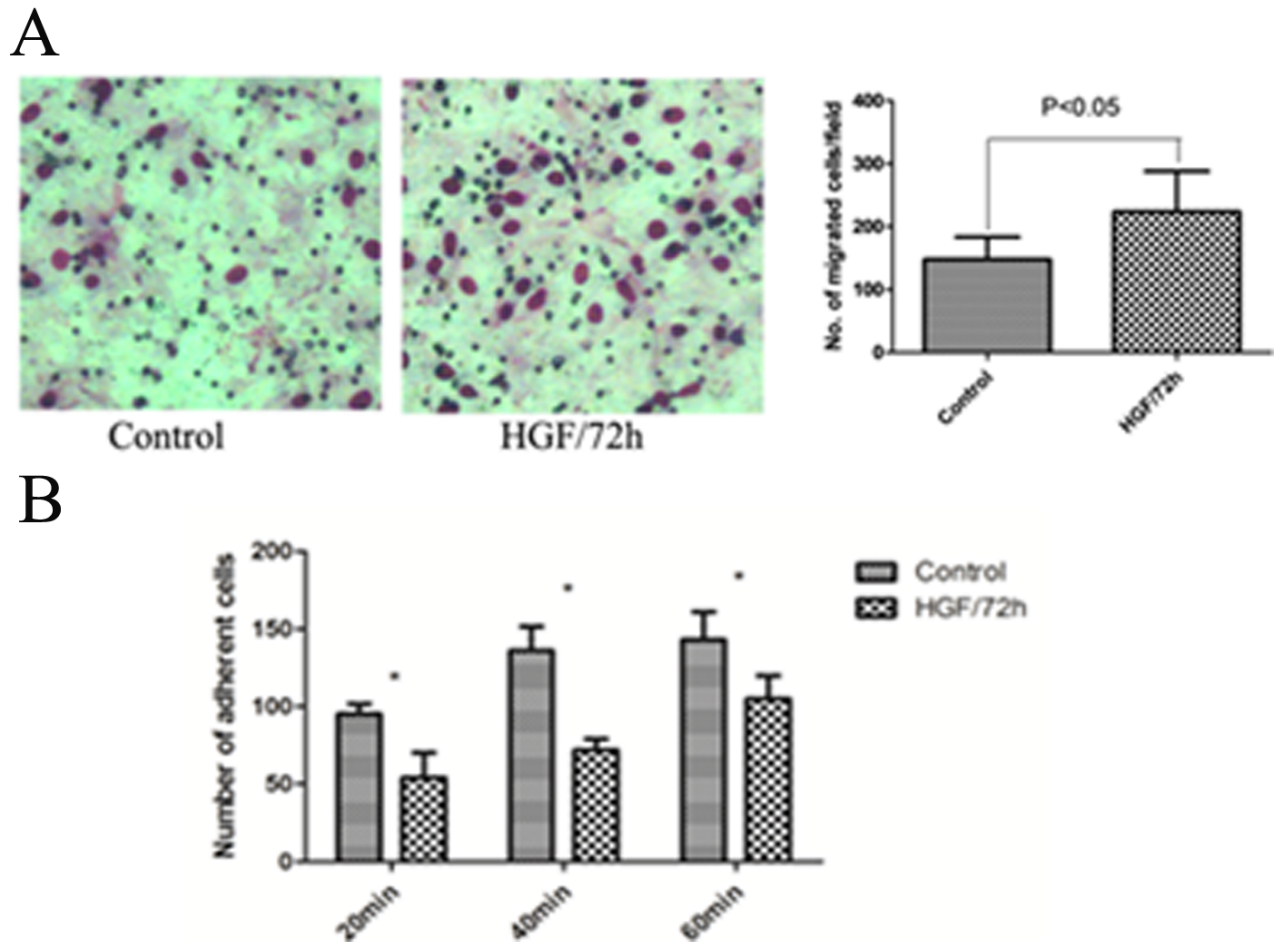
Matrigel cell invasion and adhesion assay of Huh7 cells treated with 10 ng/ml HGF for 72 h. A, The results of matrigel invasion assay. B, The count number of cell adhesion assay. All the data results are from three independent experiments.

Since the cells occurred EMT, whether its cell function had changed. Matrigel invasion assay in transwell was performed to assess the invasive ability. We compared the invasive ability of Huh7 cells with HGF-induced for 72 h to those cells treated without HGF. As shown in Figure 4A, the number of HGF-induced cells (223±60) passed through ±35). It proved that increased invasive ability of HGF-induced cells. E-cadherin can mediate adhesion between adjacent cells. Since the expression of E-cadherin was reduced and the cell invasive capacity was increased in the EMT model, we wanted to determine whether the cell adhesion was weakened. As show in Figure 4B, the number of adherent cells gradually increased with time prolonged, but the number of adherent cells treated with HGF for 72 h was significantly less than that of the control cells with significant statistical differences.

### Effects of cell surface glycan expression in HGF-induced EMT Huh7 Cell

**Figure 5:**
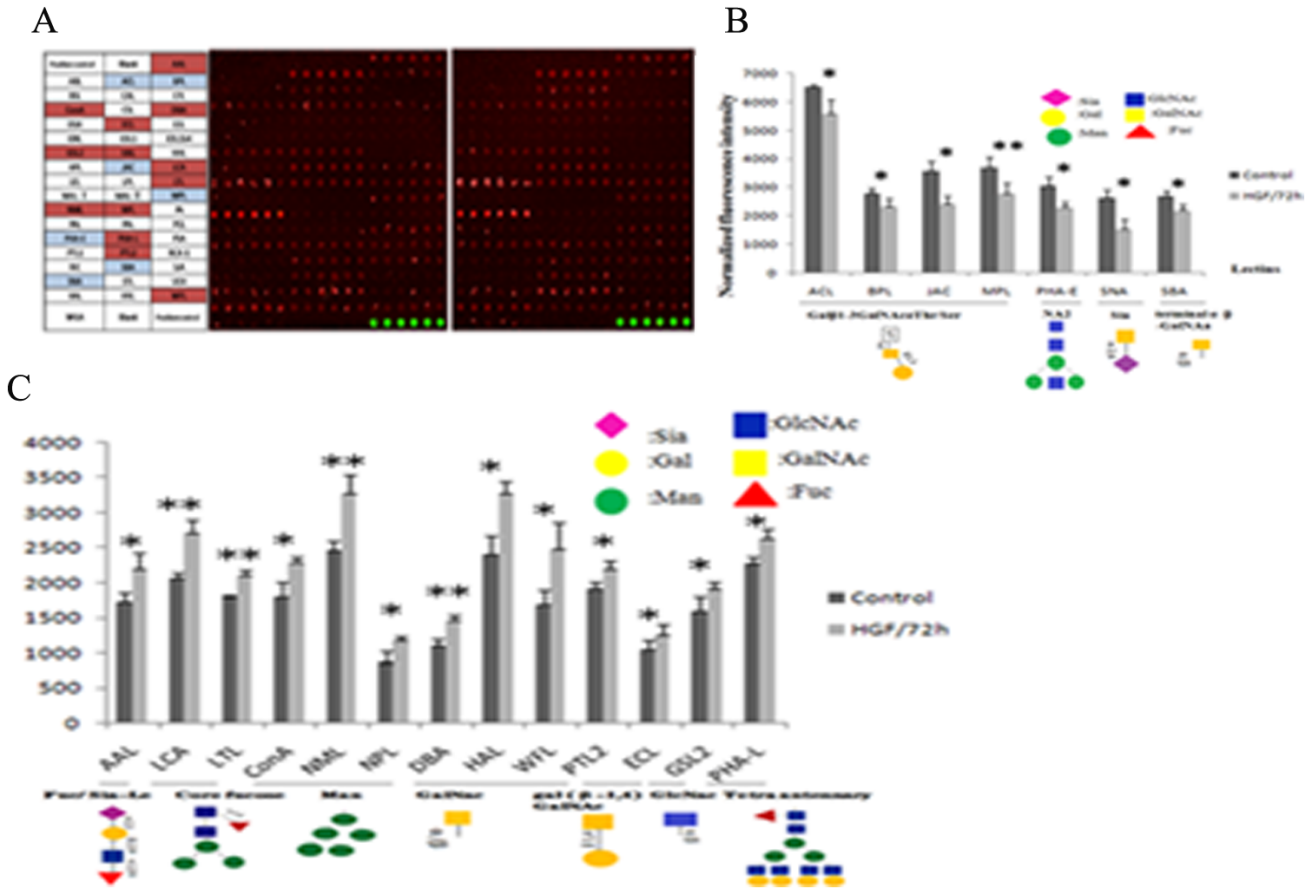
The binding affinity to surface glycan expression of Huh7 cells treated with or without HGF is analyzed by lectin microarray containing 50 kinds of lectins. A, Lectin microarray system; lectins with different binding specificities (left), bound cells on the lectin microarray were scanned with fluorescence scanner (middle and right). B, Lectins for profiling the reduced cell surface glycan expression in EMT by bar graph based on lectin microarray data. C, Lectin for profiling the raised cell surface glycan expression in EMT by bar graph based on lectin microarray data. Data are the average ± SD of three independent measurements.* represent that p<0.05, ** represent that p<0.01.

To probe the differences in glycan processing of glycoproteins in EMT, we carried out lectin microarray analysis to determine the alternation of glycan pattern of cell surface membrane glycoprotein in HGF-induced cells. Data were collected from three independent measurements, which had results with similar trends. Lectin specifically binding glycoprotein and scan images of the cells for all 50 lectins were showed in Figure 5A. Out the 50 kinds of lectins, it was discovered that the signal intensity of 20 lectins were statistically significant difference between the two types of cells. Each lectin signal was indicated in Figure 5 as normalized fluorescence intensity. To compare with the control, the binding affinity capacity of seven lectins, ACL, BPL, JAC, MPL, PHA-E, SBA and SNA, were reduced in HGF-induced EMT cells with significant difference as p<0.05 (Figure 5B). According to specific binding ability of lectins, the Galβ1-3GalNAcα-Ser (T) and GalNAcα-Thr/Ser (Tn) structures could be recognized by ACL, BPL, JAC, MPL; Siaα2-6Gal/GalNAc was distinguished by SNA; NA2 and bisecting N-acetylglucosamine oligomers (bisecting GlcNAc) could be determined by PHA-E, and terminal α or β-linked N-acetylgalactosamine was affinity binding to SBA. These glycan structures decreased substantially in HGF-induced EMT cells. Thirteen lectins, AAL, ConA, DBA, GSL II, ECL, HAL, LCA, LTL, NML, NPL, PHA-L, PTL II and WFL had enhanced their binding ability to special glycan in HGF-induced EMT cells (Figure 5C), there were statistically difference (p<0.05). According to the result of lectins-mentioned above, enhanced lectins AAL, LTL, LCA bind preferentially to terminal αFuc and ± Sia-Le, core fucose; ConA, NML, NPL bind to α-linked mannose (α-Man); ECL, PTLII, DBA, HAL, WFL prefer to bind galactosyl β (α)-linked N- acetylgalactosamine (gal-β (α)GalNAc); GSL II bind α- or β-linked N-acetylglucosamine residues (α (β) – GlcNAc), and PHA-L could specific bind the glycan with β1, 6 GlcNAc branching structures and tetraantennary complex type oligosaccharides.

**Figure 6:**
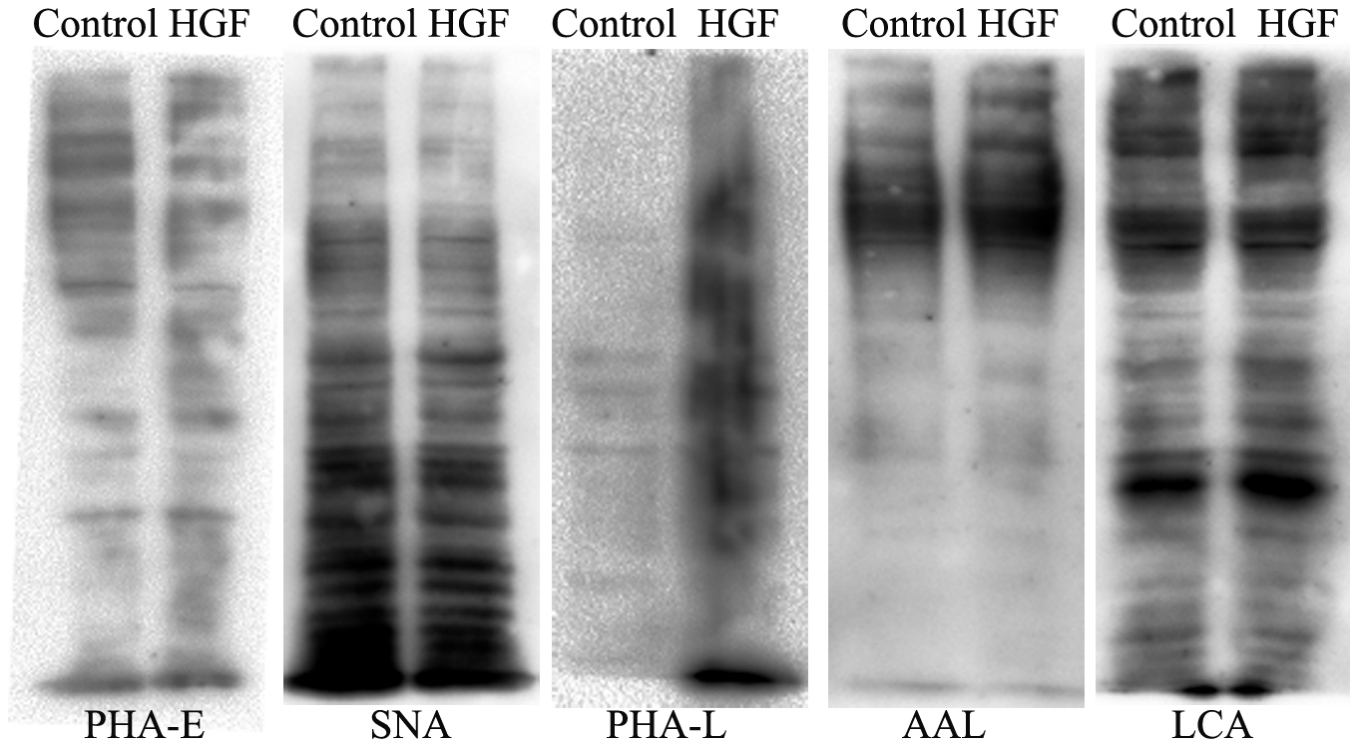
Lectin blot for biotin labeled lectins PHA-E, SNA, AAL, LCA and PHA-L.

**Figure 7:**
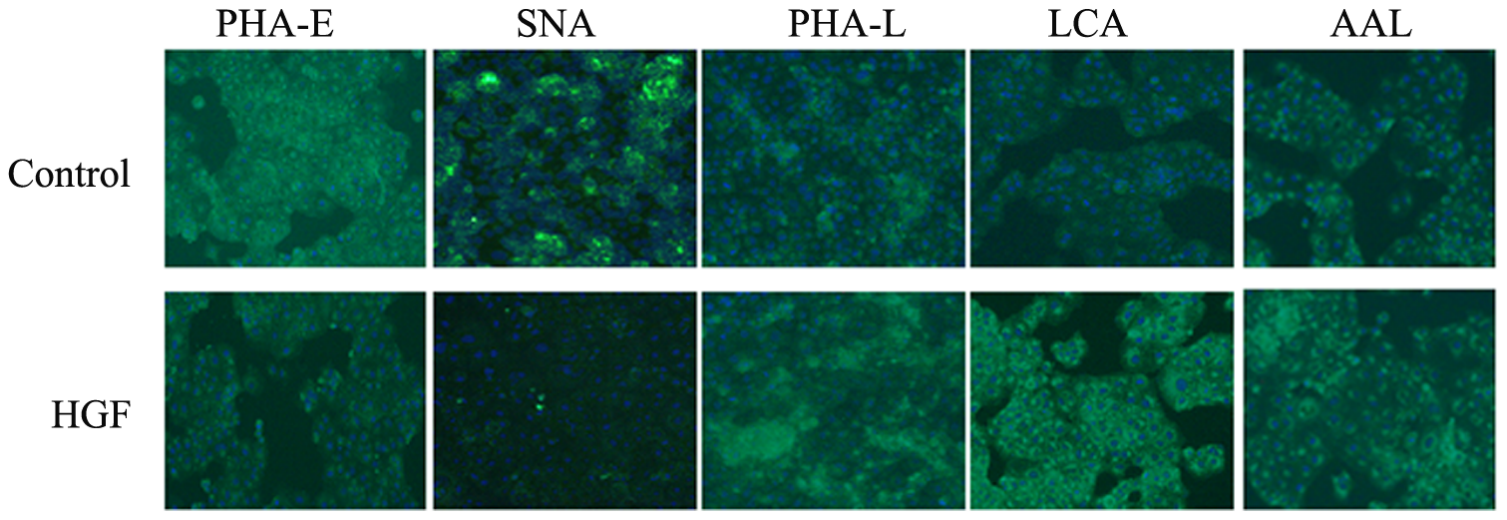
Differential glycan profiling on cell surface by fluorescence cell lectin-immunochemistry, the results were consistent with the microarray results (100×).

To validate the results of the lectin microarray, we performed lectin blot by selected biotin labeled lectins PHA-E, SNA, AAL, LCA and PHA-L. Results showed that it was coincided with the trend of lectin microarray (Figure 6). PHA-E, SNA binding glycan were decreased and AAL, LCA, PHA-L binding glycan were increased in the EMT process. The same result was achieved using fluorescence cell lectin-immunochemistry for biotin labeled lectins PHA-E, SNA, AAL, LCA and PHA-L (Figure 7). In conclusion, these two methods detected the change of glycan from cell membrane proteins and cell surface, meanwhile, verified the accuracy of the results from the lectin microarray.

### Cell surface glycan alterations in the EMT process may due to the change of glycosyltransferase expression

**Figure 8:**
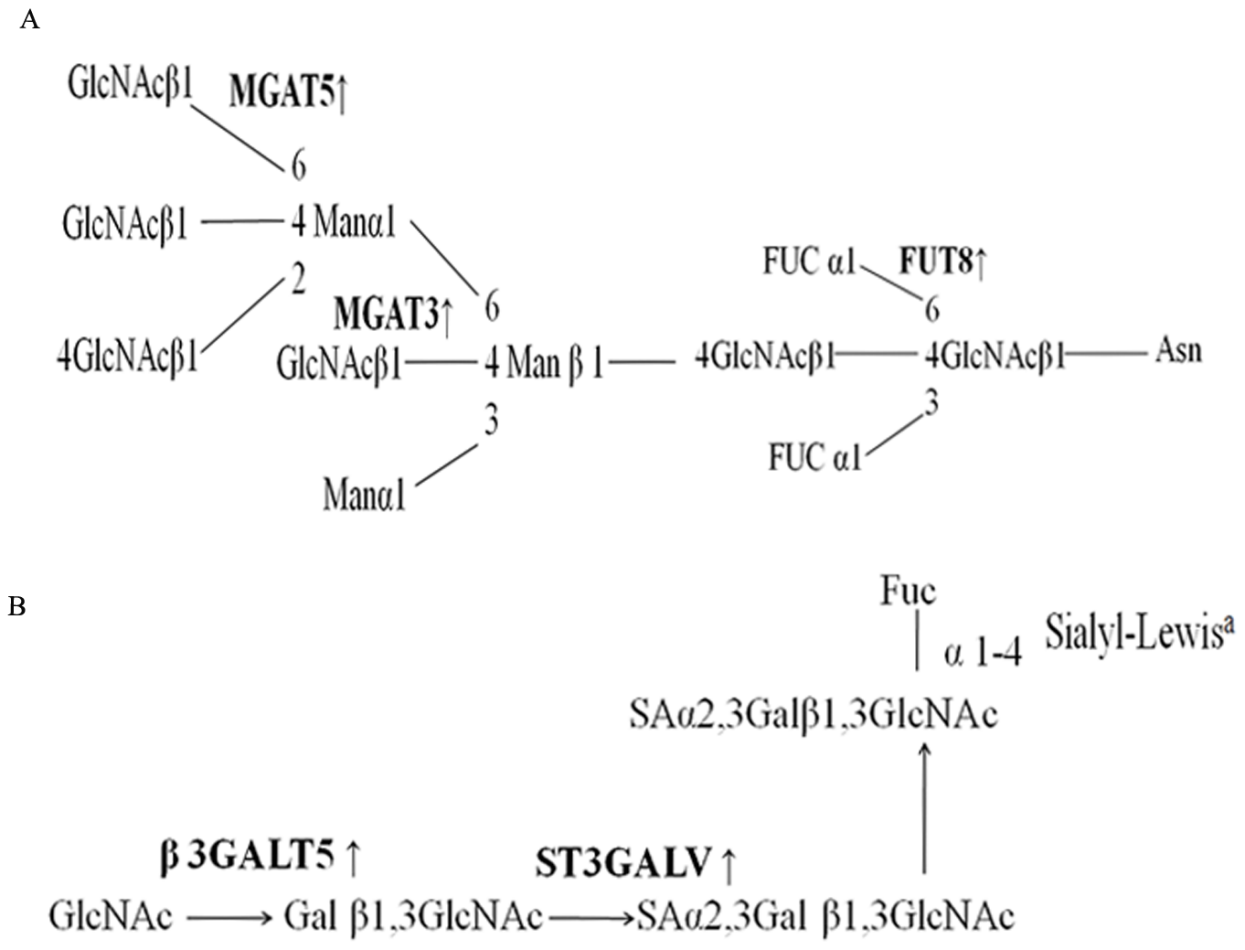
Glycosyltransferases involved in the defined glycan synthetic pathway. A, the *Mgat3*, *Mgat5* and *FuT8* involved N-glycan synthesis pathway. B, *β3GalT5* gene participate in the sLe^a^ synthetic pathway.

**Figure 9:**
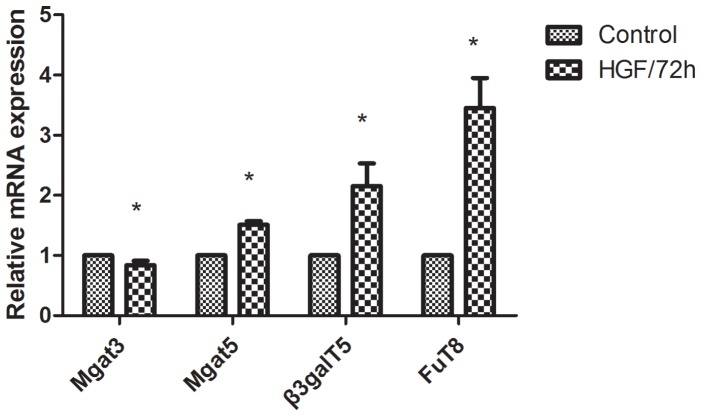
Glycosyltransferase gene expression in HGF-induced EMT Cells using a qRT-PCR analysis. *p<0.05. All the data results are from at least three independent experiments.

It was well known that glycosyltransferase gene can regulate the alteration of glycan structures resulting in a change of signal transmission. Next, we selectively analyzed the mRNA expression of 4 glycosyltransferases encoded gene to explore the enzymatic basis of the important alteration of cell surface glycan by qRT-PCR. These glycosyltransferases involved in the glycan synthesis pathway were shown in Figure 8. The experiment was executed as described in materials and methods, but the annealing temperature was 58°C. *β-actin* was used as a reference. According to previous studies, N-acetylglucosaminyltransferase III (GnT-III) which catalyzed β1, 4-bisecting- N-acetylglucosamine to N-glycan and encoded by *Mgat3* gene has been found to act as suppressors in the tumor metastasis. In contrast, N-acetylglucosaminyltransferase V (GnT-V) could add the GlcNAc β1-6 branches to N-glycan at the Man α1-6 side of the trimannosyl core and encoded by *Mgat5* gene, it was strongly associated with cancer metastasis [14]. The glycan structures catalyzed by GnT-III and GnT-V were bound by PHA-E and PHA-L respectively. Fucosylation was very common in a variety of diseases and core fucose structures catalyzed by α1, 6-fucosyltransferase (FuT8). FuT8 transfers a fucosyl residue to the asparagine-linked GlcNAc residue of complex type N-glycan, it could recognized by LCA. We have previously observed that bisecting GlcNAc was decreased and β1,6 GlcNAc branching structures and fucose were increased in HGF-induced EMT cells. To confirm whether the glycan structures alterations in EMT process was regulating by glycosyltransferase gene. *Mgat3, Mgat5* and *FuT8* mRNA expression were tested during EMT, we observed a significant decrease in *Mgat3* mRNA expression (p = 0.017), the expression of *Mgat5* and *FuT8* mRNA increased by 1.5±0.005 and 3.45±0.018 fold (Figure 9).

Human UDP-Gal: βGlcNAc β1-3-galactosyltransferase, polypeptide 5 (β3GalT5) which was a member of the β1, 3galactosyltransferase (β1, 3Gal-T) family. It is responsible to form the glycosidic bound by adding β1, 3Gal residues to GlcNAcβ1-3Galβ1-4GlcNAcβ1-R branched and then generated sLe^a^ structure which was bound by lectin AAL. Our previous study [15] found that β3GalT5 glycogene was up-regulated in high metastatic potential HCCLM3 cells by glycogene microarray. After knocking out β3GalT5, the ability of invasion and migration for HCCLM3 cells were decreased significantly. To confirm whether this phenomenon would also be observed in EMT metastatic model, *β3GalT5* mRNA was evaluated. We got the same results of *β3GalT5* mRNA increased by 2.15±0.01fold (Figure 9) in EMT metastatic model. These qRT-PCR results suggested that cell surface glycan alterations in EMT process may be based on the expression of glycosyltransferase. The result comparison of lectin microarray and qRT-PCR were listed in Table 3.

**Table 3:** The result comparison of lectin microarray and qRT-PCR.

Lectins	Glycan structure	Glycogenes
PHA-E↓	NA2 and bisecting N-acetylglucosamine oligomer	*Mgat3*↓
PHA-L↑	β1,6 GlcNAc branching structures	*Mgat5*↑
LCA↑	core fucose	*FuT8*↑
AAL↑	sLe^a^	*β3GalT5*↑

## Discussion

Recent studies discovered that EMT has a very significant connection with the cancer in situ invasion and distant metastasis, such as ovarian cancer, breast cancer, colon cancer, lung cancer, prostate cancer, liver cancer, and so on [16]. Research has revealed that EMT may be induced either by environmental stresses or by a number of extracellular mediators. HGF is a multifunctional cytokine secreted by hepatocytes, it can induce EMT that could weaken tumor cell adhesion and enhance invasion in some tumor models. Purified HGF can stimulate invasive ability in various cancer cells in vitro [17]. In the present study, we used HGF induced EMT metastasis model in Huh7 hepatocellular carcinoma cells for exploring the mechanism of HCC metastasis. As expected, the HGF induced cells became spindle shape, increased cell migration and weakened adhesion ability, and EMT-related molecular, E-cadherin decreased both in mRNA and protein level, the expressions of N-cadherin, Slug, Snail, Twist, Vimentin and α-SMA were raised after HGF stimulating, all of the changes were in line with EMT. Our data showed the same results as previous study using HGF induced EMT in HCC cell lines (17).

Cell surface glycan signature can vary during cell-cell interactions, growth regulation and differentiation. Altered glycan signatures are also observed on tumor cells, and play a critical role in the escape of tumor cells from immune surveillance as well as metastasis. Tumor cell metastasis may alter cell membrane properties determined by cell surface glycoconjugates. For example, cell surface sialylation and β1-6 branching of N-linked oligosaccharides are strongly correlated with differentiation and metastatic of cancer cells [18], [19]. There are various analytical techniques for glycan structure, such as affinity chromatography, mass spectrometry or NMR; none of them are suitable for a global survey. Lectin microarray is a novel, rapid, high-throughput and high-sensitivity glycan profiling method. Many previous studies using lectin microarray were detected “cell extracts” rather than intact cells, but the highly multivalent and diverse nature of glycan on each cell surface may be damaged during the process of cell lysates and failed to profile the intact cell surface glycan [20]. Here we used this technology to analyze the cell-surface glycan signature of whole cells directly. Cy5-labeled cells were applied in situ to the lectin microarray. After washing, bound cells were directly detected by a fluorescent scanner. This lectin microarray was established by our laboratory using gel-slide as solid support. There was a linear relationship between the fluorescence intensity and the coefficient of variation of fluorescence signal was controlled within 10%. The lectin microarray results were verified by identifying the differences in glycan chain of standard glycoprotein:asialofetuin and fetuin. This lectin microarray can identify the N-glycan compositions of alpha-fetoproteins (AFPs) from different sources [12]. The preliminary data confirms that our lectin microarray system was reliable.

Glycan remodeling is a major aspect of the cell differentiation process, immune activation. Previous studies have reported some glycan structural changes associated with cells differentiation and tumor progression. Guan found glycosphingolipids (GSLs) was down-regulated and GalNAc-type O-glycosylation was up-regulated in TGF-β induced EMT process [21], [22]. Kevin discovered 40 glycogens related with TGF-β induced EMT [23]. There poor studies systematically and exhaustively research the major glycan structural changes in cell migration. In this case, we analyzed the glycan alterations in EMT by lectin microarray and found some abnormal glycosylation structures as indicators for cell migration; the data were showed in Figure 5. A large number of studies have shown that the alterations of glycan related to HCC progress closely. We observed the cells with EMT could have more stranger affinity to PHA-L than that to PHA-E, this two lectin specific identificated β1-6 branched structure and NA2 and bisecting GlcNAc respectively. GnT-III was a key glycosyltransferase in N-glycan biosynthetic pathway and could suppress the formation of β1,6 GlcNAc branching structure catalyzed by GnT-V [24]. In a tumor context, GnT-III and GnT-V have antagonistic effect, GnT-III and its bisecting GlcNAc structure can suppress tumor metastases whereas GnT-V and its β1,6 GlcNAc branching structure increased malignancy and metastasis [25]. Previous studies had suggested that GnT-III prevented metastasis via enhance cell-cell adhesion (E-cadherin glycosylation) and down-regulated cell-ECM adhesion (Integrin glycosylation), the overexpression of GnT-III increased the bisected GlcNAc structure of E-cadherin and regulated the cell-cell adhesion of E-cadherin-mediated. Meanwhile, the bisects structure of GnT-III catalytic inhibited α3β1 and α5β1 integrin binding the laminin in extracellular matrix and involved in cell migration [26]. Pinho demonstrated that loss and recovery of *Mgat3* and GnT-III mediated E-cadherin N-glycosylation was a mechanism in EMT through TGF-β1 induce or removal initiates EMT/MET program [27]. Xu discovered that a decrease in GnT-III expression led to reduce the bisected N-glycan, but the expression of GnT-V was enhanced in the TGF-β1 induced EMT, over-expressed GnT-III can inhibited TGF-β1 induced EMT, nuclear localization of β-catenin and cell migration [28]. GnT-V was highly involved in malignant transformation of cancer and its activity was well correlated with the severity of cancer. Glycan structure generated by *Mgat5* had a high-affinity bound to galectin ligand, the increased expression of *Mgat5* brought about growth factors receptor resided on the cell surface, caused cells malignant by continuing transfer cell growth signals [29]. In GnT-V transgenic mice, GnT-V overexpression promoted EMT and keratinocyte migration by enhancing EGF receptor signaling [30]. In our studies, we captured the classic metastasis-associated carbohydrate chain structures by corresponding lectin, then lectin blot and fluorescence cell lectin-immunochemistry were both confirmed this result, which was consistent with previous research [31].

T/Tn antigens were specific tumor-associated carbohydrate antigen markers of cell surface mucin. Lectin ACL, BPL, JAC and MPL can bind this structure. Tn has a simple structure composed of N-acetyl-D-galactosamine with a glycosidic α linkage to serine/threonine residues (GalNAcα1-O-Ser/Thr) and was catalyzed by polypeptide α-N-acetylgalactosaminyl -transferases (ppGalNAcTs) in Golgi. Then Tn antigen was modified by T-synthase extended to the core 1 O-glycan Galβ1-3GalNAcα1-OSer/Thr (T antigen). Cumulative studies reported that the Tn antigen has high levels in human cancer, common in colon and breast cancer [32], [33], it also correlates with metastatic potential and poor prognosis. We found T/Tn antigens were reduced in EMT model; it provided reference data for the research in HCC and its metastasis. Many studies have demonstrated that fucosylation associated with the biological aggressiveness of tumor. α-1, 6 fucosylation is a significant glycan modification of HCC metastasis. Fucosylated AFP, AFP-L3 as a specific marker was used to diagnosis HCC [34]. The finding that AAL, LTL and LCA lectin signals were enhanced in the EMT model prompted an increase in fucosylation and Sia-Le during HCC metastasis, a result consistent with those from the lectin blot and fluorescence cell lectin-immunochemistry. Further studies implied that the up-regulation of fucosylation may be due to the increased of FUT8 expression in the HCC EMT metastasis model. Dai et.al [35] found that core fucose extent increased with hepatoma cells metastatic potential. Studies have shown that FUT8 can promote tumor cell proliferation, invasion and metastasis and is closely related to the occurrence and development of HCC. β3Gal-T5 was required for the synthesis of the Sialyl Lewis a antigen (sLe^a^), the epitope of CA19-9, which is a well-known tumor marker for gastrointestinal and pancreatic cancers. SLe^a^ is associated with the adhesion and invasion of tumors, sLe^a^ on the cell surface are associated with the dissemination of the tumors as it can recognize E-selectin of vascular endothelial cells and mediate the adherence of tumor cells to vascular endothelial cells. A higher level of expression of sLe^a^ indicates stronger tumor invasion [36]. Consistent with previous study [15], the *β3GalT5* glycogene was increased in the HCC EMT model; it implied that *β3GalT5* and its product may play an important role in the development and metastasis of HCC.

We also used lectin microarray technology to discover that the Siaα2-6Gal/GalNAc and terminal α or βGalNAc structures were down-regulated, and that the structures of mannose, gal-β (α) GalNAc andα (β) – GlcNAc were up-regulated in the HCC EMT metastasis model. Consistent with the previous result, the Siaα2-6Gal/GalNAc was reduced in HCC with metastasis than in HCC without metastasis [31], and there was an increased GlcNAc structure in highly metastatic HCCLM3 [37] and serum glycoprotein of HCC with metastatic [38]. There are also reports of increased mannose structural modification in human and rat HCC GGT [39]. On the contrary, sialic acid changes have been reported in many tumor metastases, its increases may reduce tumor cell adhesion to extracellular matrix. The causes of inconsistent results regarding the cell EMT transformation mechanism are not entirely clear, as many factors are involved in this process. Future work will aim to identify the glycoprotein of the structures identified here by lectin microarray and seek to determinate biosynthetic pathways and the mechanism of glycan alterations in EMT.

This is the first study to analyze the nature of glycan alterations in cancer EMT using intact cell surface glycoprotein by lectin microarray. The T/Tn-antigen, NA2 and bisecting GlcNAc, Siaα2-6Gal/GalNAc, terminal α or βGalNAc structures were reducing markers, while terminal αFuc and ± Sia-Le, α-or β-linked GalNAc, core fucose, β1,6 GlcNAc branching structures and tetraantennary complex oligosaccharides were increasing markers for HCC EMT. The specific genes and proteins which control these glycan structures may be targets to prevent or reverse EMT. These findings provide important information for future research on the prevention of HCC metastases and drug resistant forms of HCC cells.
